# Neuropharmacological and Antidiabetic Potential of *Lannea coromandelica* (Houtt.) Merr. Leaves Extract: An Experimental Analysis

**DOI:** 10.1155/2022/6144733

**Published:** 2022-03-28

**Authors:** Fahadul Islam, Saikat Mitra, Mohamed H. Nafady, Mohammad Tauhidur Rahman, Vineet Tirth, Aklima Akter, Talha Bin Emran, Amany Abdel-Rahman Mohamed, Ali Algahtani, Sanad S. El-Kholy

**Affiliations:** ^1^Department of Pharmacy, Faculty of Allied Health Sciences, Daffodil International University, Dhaka 1207, Bangladesh; ^2^Department of Pharmacy, Faculty of Pharmacy, University of Dhaka, Dhaka 1000, Bangladesh; ^3^Faculty of Applied Health Science Technology, Misr University for Science and Technology, Giza 12568, Egypt; ^4^Department of Cardiology, Z.U Model Hospital, Feni 3900, Bangladesh; ^5^Mechanical Engineering Department, College of Engineering, King Khalid University, Abha 61421, Asir, Saudi Arabia; ^6^Research Center for Advanced Materials Science (RCAMS), King Khalid University, Guraiger, Abha 61413, Asir, P.O. Box No. 9004, Saudi Arabia; ^7^Department of Pharmacy, BGC Trust University Bangladesh, Chittagong 4381, Bangladesh; ^8^Department of Forensic Medicine and Toxicology, Zagazig University, Zagazig 44511, Egypt; ^9^Department of Physiology, Faculty of Medicine, Kafrelsheikh University, Kafrelsheikh, Egypt

## Abstract

The present study examines the neuropharmacological and antidiabetic properties of methanol leaves extract of *Lannea coromandelica* in animal models. This study is carried out by elevated plus-maze apparatus, motor coordination, thiopental sodium has an induction role in sleeping time, hole board, hole cross, open field, antidiabetic studies. Mice were treated doses of 100, 150, and 200 mg/kg body weight in elevated plus-maze apparatus and motor coordination; 100 and 200 mg/kg body weight in sleeping time, hole cross, hole board, and open field tests; and 200 and 400 mg/kg body weight in the antidiabetic activity test. Extraction specifies a significantly decreased time duration and sleeping time in a thiopental sodium-induced sleeping time test. The experimental extract decreased locomotor and exploratory behaviors of mice in the open-field and hole-cross tests compared to the effects of the control. Furthermore, the extract increased sleeping time with a dose-dependent onset of action. The hole-board test extract also demonstrated a reduced number of head dips. The findings showed that *L. coromandelica* has potential neuropharmacological effects. In addition, in alloxan-induced diabetic mice, leaves extract at 200 and 400 mg/kg body weight revealed significant antidiabetic properties and could be used to manage blood glucose levels with more research.

## 1. Introduction

A stressful lifestyle has recently been connected to several neurological disorders, indicating that depression is an apparent medical problem in these disorders [1–4]. Antipsychotic agents such as tricyclic antidepressants (TCAs), monoamine oxidase inhibitors (MAOs), and selective serotonin reuptake inhibitors (SSRIs) are available to treat various psychotic disorders. Still, their long-term employment is being hindered by several unwanted side effects [5, 6]. Therefore, exploring novel anxiolytic agents with low detrimental consequences is still ongoing for a suitable pharmacological-related influence. Depression, for instance, is a frequent but severe kind of mental illness that causes significant emotions such as sorrow, loss of interest, influencing sleep, nutritional intake, intellectual, and psychomotor-guided everyday behaviors and activities. Depression, sadness, and anxiety can exacerbate symptoms, worsen concerns, and additionally increase the risk of suicide tendency [7, 8]. Depression and anxiety have an etiology that is, at this time, obscure. However, these could occur due to antioxidant protection system disturbance and an increased oxidation-reduction disparity or oxidative stress [9, 10]. The brain can be affected by redox stress that plays a role in accelerating the cellular disorders that lead to neurological destruction and mental decline. Sedatives and hypnotics are commonly used to alleviate anxiety as they induce a relaxing potential to induce sleep and maintaining sleep duration. Clinically indicated antidepressants include TCAs, SSRIs, and specific serotonin–noradrenaline reuptake inhibitors (SNRIs). These drugs are now widely utilized in the treatment of various psychiatric conditions. However, major side effects such as immune system dysfunctions, as well as declines in cognitive performance, physical dependency, and intolerance have contributed to the continued use of these currently accessible synthetic drugs [11–14].

Diabetes mellitus, or simply diabetes, is a syndrome characterized by high blood glucose levels that result from defects in the body's ability to produce and/or use insulin. About 422 million people worldwide have diabetes, particularly in low- and middle-income countries, making diabetes mellitus one of the leading causes of death [15, 16]. Chronic hyperglycemia during diabetes causes the glycation of proteins, which in turn leads to complications (damage), affecting the eyes, kidneys, nerves, and arteries [17]. Given the important role of oxidative stress in the pathogenesis of many clinical conditions and aging, antioxidant therapy could positively affect the natural history of several diseases. Hyperglycemia activates a particular metabolic route that involves diacylglycerol, protein kinase C, and NADPH-oxidase, culminating in reactive oxygen species (ROS) [18]. Hyperglycemia in people with diabetes is thought to cause ROS to be produced by lipoxygenases, cyclooxygenases, nitric oxide synthases, and peroxidases [19, 20].

Plants are a vital source of preventive and therapeutic bioactive molecules, which contribute as an essential regimen in traditional and modern medicine [21, 22] People around the globe have long used plant-derived products to relieve certain illnesses [23, 24] *Lannea coromandelica* (Houtt.) Merr is a deciduous tropical tree found in India, Bangladesh, and other tropical countries, belonging to the Anacardiaceae family. From the stem barks of *L. coromandelica*, five dihydroflavonols were extracted and characterized [25]. Ulcers, wounds, ophthalmia, gout, sprains, diarrhea, ulcerative stomatitis, and dysentery gain from the bark, whereas elephantiasis, inflammation, neuralgia, sprains, and bruises benefit from the leaves [26]. The anti-inflammatory [27], hypotensive [28], and cytotoxicity effects of the extract of *L. coromandelica* stem barks were investigated [29]. Plants' wound-healing abilities have been studied in folklore since then. The significant accomplishments have prompted more studies into medicinal plants to verify these well-known properties. *L. coromandelica* was proven to be effective in the healing process in this investigation. The extracts' ointment was made and tested on several models for wound-healing properties. Microbial infection can stymie the healing process of a wound. According to studies, several plants utilized for wound treatment have operational standards or elements with antibacterial properties [30].

The present study examined the neuropharmacological and antidiabetic properties of the methanol leaves extract of *L. coromandelica*. The results demonstrate the significance of the leaves for potential use in common mental illnesses related to depression as well designed to stimulate specific antidiabetic targets.

## 2. Materials and Methods

### 2.1. Plant Material and Chemicals

Thiopental sodium, diazepam, and glibenclamide were purchased from Incepta Pharmaceuticals Ltd. Bangladesh. Various reagents and distilled water were purchased from the British Drug House (BDH) Chemicals Ltd. The *L. coromandelica* plant material was collected from Koyra, Khulna. The genus and the plant family were identified from National Herbarium, Bangladesh. (DACB accession number: 35242).

### 2.2. Methanol Extract Preparation

The leaves collected from *L. coromandelica* were separated from unwanted materials and washed with distilled water. The leaves were shade-dried and ground into a coarse powder using a processor. Before the study was initiated, the powder-filled container was stored in a dark, calm, and dry environment. A quantity of 400 gm granulated leaves of *L. coromandelica* was bathed in 1000 mL of 95% methanol for ten days in a glass-looking flask with constant vibration and stirring. Subsequently, the entire blend was purified across a fine, white cotton material and Whatman filter paper No. 1 to obtain a clear filtrate. The filtrate was placed in an open room to dissolve the solvent, and the extract was achieved. The yield was found to be 2.07% w/w freshly prepared extract was used in the experiments..

### 2.3. Experimental Animals

Swiss Albino mice (*n* = 180) of both sexes (22–25 gm) were bought from Jahangirnagar University in Dhaka, Bangladesh, and maintained in animal cages under 22–25°C, moistness 60–70%, light for 12 hours, and dull cycle for 12 hours. A regular pellet diet was fed to the mice. Authorized methods were used in this study, involving the use of animals by The Allied Health Sciences Faculty represented by its Committee for Research Ethics, Daffodil International University, Dhaka 1207, Bangladesh, with Ref. No.: FAHSREC/DIU/2020/1007(5).

### 2.4. Phytochemical Group Screening

The presence of some phytochemical groups was examined in the preliminary phytochemical study. Steroids, gums, saponins, tannins, alkaloids, flavonoids, cardiac glycosides, and terpenoids were detected by colorimetric methods [31] (Table 1).

### 2.5. Acute Toxicity Test

The extract was given orally to groups of mice (*n* = 5) in dosages of 100, 200, 400, and 600 mg/kg, and percentage fatality was measured from 24 hours to 7 days [32].

### 2.6. Anxiolytic Activity Assessment in Mice Utilizing the Elevated Plus-Maze

The elevated plus-maze is considered a fundamental apparatus for studying practically all antianxiety drugs [33]. The maze a, 40 cm walls in height and contains two opposed open arms (50 cm × 10 cm) intersected by two enclosed arms. The arms were joined with a central square measuring 10 cm by 10 cm to provide a plus sign presence. The maze was raised about 70 cm in a bad lighting area over the ground. Rodents have a genuine distaste to high and open places, desiring confined arms with a burrow-like atmosphere and hence spend more time in enclosed arms. When the mice are presented to the new maze alley, they participate in an approach-avoidance conflict greater in the open arm than in the confined arms. The mice were placed into five groups, with five in each group. The mice were given the following treatments: distilled water (10 mL/kg, p.o.), leaves extract (100, 150, and 200 mg/kg, p.o.), and diazepam (1 mg/kg, p.o.).

### 2.7. Influence on Motor Management

The mice were prepared to be stable for two minutes on a spinning rod at 20 rpm speed [34]. Only mice that could be stable were selected for the experiment. Every mouse was positioned on the rotarod separately, and within 2 minutes, a whole number of falls was recorded, which was employed to determine the basal reading. The mice were grouped into five groups, with five animals each. The mice were contained on the rotarod one hour after having distilled water (10 mL/kg, p.o.), leaves extract (100, 150, and 200 mg/kg, p.o.), and diazepam (1 mg/kg, p.o.).

### 2.8. Test of Thiopental Sodium-Induced Sleeping Time

Anisuzzman et al. [35] studied the leaf extract activity to test the sleeping time stimulated by thiopental sodium. In this case, mice were distributed into four groups with five animals in each group. The control group was group I and was provided with distilled water orally and diazepam (0.5 mg/kg, b.w., p.o.). It was used as a standard and was given to group-II orally. At prescribed amounts of 100 and 200 mg/kg body weight, Group III and IV, respectively. After half an hour, intraperitoneally, thiopental sodium (20 mg/kg b.w.) was administered to all studied groups for inducing sleep. Due to uncoordinated movements, separate mice have been put on a table and administered separately. By utilizing the following formula to determine the effect percentage:

(1) $$\begin{array}{c} \text{effect}\left(\%\right)=⟶\frac{\text{average duration of loss of righting reflex in the test group}}{\text{average duration of loss of righting reflex in the control group}}\times 100. \end{array}$$

### 2.9. Test of Hole Cross

As previously mentioned [36], the study used a cage with a 0.30 × 0.20 × 0.14 m with a partition in the center of the enclosure. In the middle of the frame, a hole was created with a diameter of 0.03 m at the height of 0.075 m. Experimental animals were classified into different groups and positioned on one side of the frame. After processing the control, standard, and test extracts (p.o.), the number of mouse passages across the hole from one chamber to another was calculated for 3 minutes at 0, 30, 60, 90, and 120 minutes. The control group was group I (provided with distilled water) and group II was recognized as a standard (provided diazepam 1 mg/kg, b.w., p.o.). At doses of 100 and 200 mg/kg b.w, groups III and IV obtained the methanol leaves extract:

(2) $$\begin{array}{c} \text{movements inhibition}\left(\text{\%}\right)=\frac{\text{mean no}.\text{of movements}\left(\text{control}\right)-\text{mean no}.\text{of movements}\left(\text{test}\right)}{\text{mean no}.\text{of movements}\left(\text{control}\right)}\times 100. \end{array}$$

### 2.10. Test of Hole Board

The process was carried out with minor differences as stated by Kamei J et al. [37]. The present research was conducted on a 90 cm × 90 cm radius platform and 16 equally spaced holes. The structure of this platform was also 5 cm high. Study groups were divided with control, standard, and test mice. Each study group has five mice (*n* = 5). The control group, Group I (10 mL/kg b.w.), was provided distilled water. Diazepam (1 mg/kg, *b*. w., p. o.) was introduced to group II as a standard. Groups III and IV were provided the extract in 100 and 200 mg/kg body weight doses, respectively. The number of head dips into the holes was calculated 10 minutes later to the application.

(3) $$\begin{array}{c} \text{Inhibition }\left(\%\right)=\frac{\text{mean no.of head dips}\left(\text{control}\right)-\text{mean no.of head dips }\left(\text{test}\right)}{\text{mean no.of head dips}\left(\text{control}\right)}\times 100. \end{array}$$

### 2.11. Test of Open Field

This study was conducted using the schemes were introduced by Gould TD et al. [38] A 0.5 m^2^ regular field with a square trend makes up the test device. Moreover, squares with black and white colors have been formed. The experimental board seems like a chessboard in structure. Likewise, the automated operation system received a section height of 0.1 m. A set of four groups of mice were formed. Each group consisted of five mice (*n* = 5). Group I was assigned to the control group and given distilled water (10 mL/kg b.w.). Group II received the standard dose of diazepam (1 mg/kg, b.w., p.o.). Groups III and IV received leaves extract doses of 100 and 200 mg/kg body weight, respectively. At time points of 0, 30, 60, 90, and 120 minutes after the oral route of the test drug, the number of the square traveled at every speed by the mice in every considered group was recorded for 3 minutes.

(4) $$\begin{array}{c} \text{Inhibition}\left(\%\right)=\frac{\text{mean no.of movements}\left(\text{control}\right)-\text{mean no.of movements }\left(\text{test}\right)}{\text{mean no.of movements}\left(\text{control}\right)}\times 100. \end{array}$$

### 2.12. Experimental Design

The mice were divided into five groups, each containing five animals. Group I was a normal control (saline, nondiabetic), group II was an alloxan (150 mg/kg)-treated control (diabetic control), group III was given Glibenclamide (10 mg/kg), groups IV and V were given leaves extract at doses of 200, and 400 mg/kg, respectively, treatment sessions lasted 15 days in a row. Oral gavage was used to give the extracts and saline solution.

### 2.13. Antidiabetic Activity

An in vivo oral glucose tolerance test was used to evaluate the antidiabetic potential of extract using albino mice at doses of 200 and 400 mg/kg body weight. The doses of 200 and 400 mg/kg body weight were chosen based on their conventional assertion efficacy [39].

### 2.14. Blood Glucose Determination

The blood glucose level of each mouse was determined by utilizing a fractional tail amputation procedure and a one-touch electronic glucometer with glucose test strips to draw blood from the tail vein. To protect the tails from infection, they were washed with ethanol [40].

### 2.15. Body Weight Analysis

The experimental mice body weight is measured before they began the medication (day 0) and throughout the trial (days 7 and 15), as well as fluctuations in weight.

### 2.16. Statistical Analysis

The data analysis has been calculated from utilizing version 20 of SPSS statistical software. Results were expressed as the mean ± SEM (standard error mean) value. Moreover, the one-way analysis of variance (ANOVA) accompanied by Dunnett's test for elevated plus-maze apparatus, motor coordination, sleeping time, hole board, and antidiabetic tests were utilized. Two-way ANOVA followed by Bonferroni's tests was implemented for the hole cross and open field tests. ^*∗*^*P* < 0.05 and ^*∗∗*^*P* < 0.01 were reported to be statistically significant.

## 3. Results

### 3.1. Phytochemical Group Test

### 3.2. Acute Toxicity

Normal behavior was observed in mice given the extract at doses of 100–600 mg/kg p.o. They were awake and aware, with typical combing, touching, and pain responses. Passivity, stereotypy, or vocalization was not present. They had typical motor behavior and secretory indications. There was no evidence of depression in the mice. The animal's vigilance, motor function, limb tone, grip strength, and locomotion were normal. The extract's safe form was determined up to 600 mg/kg in mice.

### 3.3. Role of Using the Elevated Plus-Maze Apparatus in the Evaluation of Anxiolytic Activity in Mice

Regrading to the arm's open form and their height (70 cm) from the floor, with two open and two enclosed arms, the elevated plus-maze prompted a unique environment that stimulates anxiety in animals. When the animals were positioned on the maze, they desired the enclosed (dark) arms. They demonstrated anxiety and fear-like behaviors such as rigidity, freezing, and feces when they moved into the open arms. When associated with the control group, the methanol extract has been extracted at the dose's levels (100, 150, and 200 mg/kg, p.o.). This section had a significant boost in percent preference for an open arm as the first entry, the sum of transactions in the open arm, or the length of time spent in the open arm (Table 2). Diazepam (1 mg/kg, p.o.) raised the percent preference and total number within the open arm and the time of residence in the open arms significantly (^*∗*^*P* < 0.05, ^*∗∗*^*P* < 0.01), demonstrating anxiolytic effects.

### 3.4. Influence on Motor Coordination

After treating the animals with methanol leaves extract at the dose's levels of 100, 150, and 200 mg/kg, there was a significant boost in the frequency of falls within 2 minutes (Table 3). Compared to the control group, the diazepam-considered group experienced more significant falls.

### 3.5. Test of Sleeping Time

In the procedure of the thiopental-induced hypnosis, extract at doses of 100 and 200 mg/kg demonstrated a substantial decrease in the period to sleep onset in a dose-dependent way, primarily with methanol extract of *L. coromandelica* leaves (Table 4). The extract generated outcomes equivalent to the standard drug diazepam at sleep onset. The period of thiopental sodium impacted sleeping time in laboratory mice and was potentiated by extract doses compared to controls.

### 3.6. Test of Hole Cross

Compared to the control group's mice, the number of the holes traveled from one space to another in the hole cross setup was observed in periods ranging from 30 to 120 minutes. The leaves extract was found to have a constant decrease in activity in the laboratory mice at 100 and 200 mg/kg doses. In dose-dependent terms, efficient ^*∗*^*P* < 0.05, ^*∗∗*^*P* < 0.01) findings have been produced (Table 5). The fifth phase of this research revealed that leaves extract at 200 mg/kg suppressed locomotor function revealed 60%. Diazepam suppressed the behavior by 60% in the identical experimental procedure in this experiment.

### 3.7. Test of Hole Board

Using the hole-board test, the methanol extract at 200 mg/kg b.w. induced 44.64% inhibition of movement higher than diazepam, which recorded 50.89% inhibition (Table 6).

### 3.8. Open Field Test

At doses of 100 and 200 mg/kg b.w., experimental extract considerably decreased function of locomotor in mice (^*∗*^*P* < 0.05, ^*∗∗*^*P* < 0.01, ^*∗∗*^*P* < 0.01) apparent from the 1st observation (0 min) and continued up to the final observation (120 min) (Table 7). Moreover, diazepam showed a substantial reduction in locomotion in mice from the 2nd observation to the last observation. In this experiment, the methanol extract demonstrated a maximum of 44.03% inhibition of locomotor activity, while diazepam showed 54.47% inhibition.

### 3.9. Antidiabetic Activity

#### 3.9.1. Blood Glucose Level

The leaves extract of *L. coromandelica* administered at 200 and 400 mg/kg considerably lowered the blood glucose level in diabetic mice at the end of the trial in a dose-dependent manner, but not as much as Glibenclamide-treated mice (Table 8).

#### 3.9.2. Body Weight Changes and Fasting Blood Glucose Levels

After 15 days of therapy, the leaves extract at doses of 200 and 400 considerably enhanced body weight and induced a maximum decline in fasting glucose levels in alloxanized diabetic mice (Table 9).

## 4. Discussion

Since ancient times, natural remedies derived from various medicinal plants have been employed for their therapeutic characteristics [41, 42]. Organic materials are frequently used in medication, nutritional, and food additive enterprises to make herbal medications, minerals, nutritional supplements, and ailment medications. Several plant extracts have served as a source of easily available, cost-effective, and effective medication. Several ethnomedicinal herbs have been prompted to equip a neurobehavioral state as well as to control sugar level and operate as contemporary medicine alternatives [43, 44]

In open-field and hole-cross tests, the sedative effects of *L. coromandelica* were tested by monitoring the naturalistic locomotor behavior of mice. In these experiments, the duration and frequency of motion can be mitigated by agents with sedative properties, illustrated as a reduction in the curiosity of the unfamiliar setting. The hole board test was developed to assess mice reactions to a new environment and is designed to check anxiolytic-like behavior. Nonetheless, another research has found that an animal's head dipping movement is linked to their mental condition. Depending on this discovery, the anxiolytic disease corresponds with mice's anxiolytic properties that promote head dipping. At higher concentrations, 200 mg/kg, p.o. leaves extracts induced a stronger proclivity for head dipping in the mice [45]. The oppressive performance was exhibited at 30 minutes when the extract was provided and continuously for 120 minutes.

In the open-field test, the extract at the examined doses provoked considerable locomotor inhibition, which continued from 30 to 120 minutes during the studied time. The open-field test was used to evaluate the anxiety by reading the length traveled; the sleeping time was 6–12 seconds ranging, the period consumed in center squares went between 3 minutes, and the distance traveled within the center squares varied between 8.5 m^2^–19.3 m^2^.

The present study found that the extract decreased locomotor function, indicating CNS depressive properties. Both experiments resulted in a considerable decrease in mobility in mice. The extract substantially affected the mice's exploratory behavior in our studies elevated plus-maze model. Anxiolytic medications improve the time spent exploring the open arm and the number of admissions into the open arm by reducing anxiety. The plant extracts produced significant effects in mice. As observed with benzodiazepines, most anxiolytics impair memory and are often used as anxiolytics [46].

The plus-maze model has lately been utilized to research learning and memory processes in rodents, which is essential to emphasize. The depreciation of cognitive performance triggered by scopolamine, an anticholinergic drug is manifested in the delayed transfer expectancy from the open arm to the closed arm [47].

Typical drugs' therapeutic efficacy may be due to a mixture of ingredients that act as an adjuvant to the standard procedure. Many investigations have found that phytochemical substances, such as tannins, have psychoactive properties and produce nonspecific CNS depression. Gamma amino butyric acid (GABA) is a major inhibitory neurotransmitter in the CNS that has been linked to a variety of physiological processes as well as psychiatric and neurological problems. Eclectic medications have the potential to change the GABA system at the manufacturing level by stimulating GABA-mediated postsynaptic suppression via allosteric alteration of GABA receptors. With concomitant suppression of the voltage activated Ca^2+^ channel, it can either enhance chloride conductivity or enhance GABA-induced chloride conduction [48, 49]. Stimulation of protein kinase C, neuroprotective effects against ROS; boosting nicotinic receptors that also enhance perception and cognition; energetic and continuing to improve the function of the nervous; enabling the passing receptor calcium channels in the nerve cell membrane that have neuropharmacological influences are some of the suggested modes of action for alkaloids, flavonoids, steroids, and terpenoids [50–56]

According to our findings, the leaves extract of *L. coromandelica* shows potent antidiabetic properties. Methanol leaves extract of *L. coromandelica* showed significant antihyperglycemic activity in alloxan-induced hyperglycemic mice with no change in body weight; they can also enhance the effect of diabetic mellitus as demonstrated by body weight, and blood glucose level. Many mouse models have been used to study *β* cell renewal in diabetes. The balance between *β* cell renewal and loss is reflected in the overall cell mass. It was also proposed that regrowth of islet *β* cells following alloxan destruction could be the fundamental reason of alloxan-injected guinea pigs' recovery from the drug's effects [57]. After 15 days of therapy, it was observed that the leaves extract at a high dose (400 mg/kg) is more efficient than at a low dose (200 mg/kg). As a result of the foregoing explanation, leaves extract at large doses (400 mg/kg) is more effective and has a similar therapeutic effect as the standard, Glibenclamide (10 mg/kg). This could be because certain *β*-cells are still alive and active, allowing *L. coromandelica* leaves extract to exercise its insulin-releasing function.

## 5. Conclusions

The findings were all dose-dependent and statistically significant. Based on the evaluation of the current analysis, the extracts of *L. coromandelica* hold significant neuro-modulatory properties. The leaves extract had no significant effect on normal blood sugar levels, but they did effectively correct alloxan-induced alterations in blood sugar and pancreatic beta-cell population. It also had a preventive role when provided before the administration of alloxan. The activity of leaves extract on pancreatic beta-cells, as well as the lack of acute toxicity, may provide diabetics with an additional hope for future. To discover the exact phytoconstituent(s) accountable for the antidiabetic activity, more research is needed.

## Figures and Tables

**Table 1:** Phytochemical compounds of *L. coromandelica* leaves extract.

Compounds	Methanol extract
Alkaloids	++
Flavonoids	+
Saponins	_++_
Tannins	+
Steroids	++
Gums	+
Cardiac glycosides	+
Terpenoids	+

Tests were conducted in triplicates; ++ = highly identified; + = less identified.

**Table 2:** Impacts of *L. coromandelica* leaves extract and diazepam on anxiety generated by the elevated plus-maze apparatus.

Group	Dose (mg/kg)	Preference (%) open arm	Time spent (s) open arm	No. of entries open arm
Control	10 mL/kg	16.50	43.14 ± 8.88	1.97 ± 0.29
Methanol extract	100	48.0^∗∗^	63.66 ± 10.64^∗∗^	2.00 ± 0.30^∗∗^
Methanol extract	150	16.50	63.00 ± 12.90^∗∗^	2.91 ± 0.35
Methanol extract	200	37.0^*∗*^	61.80 ± 5.88^∗∗^	2.00 ± 0.33^∗∗^
Diazepam	1	66.66^∗∗^	106.86 ± 5.91^∗∗^	4.78 ± 0.38^∗∗^

Results are expressed as mean ± SEM (*n* = 5). ^*∗*^*P* < 0.05 and ^*∗∗*^*P* < 0.01 which are in contrast to the control group, are significant (one-way ANOVA followed by Dunnett's test).

**Table 3:** Effects of the *L. coromandelica* leaves extract and diazepam on the function of muscle relaxant in mice, examined utilizing rotarod apparatus.

Group	Dose	Number of falls in 2 min	
		Basal reading	After treatment
Control	10 mL/kg	6.00 ± 0.21	6.2 ± 0.37
Diazepam	1	7.00 ± 0.14	14.00 ± 0.51^∗∗^
Methanol extract	100	7.00 ± 0.21	8.12 ± 0.47^*∗*^
Methanol extract	150	8.00 ± 0.44	10.10 ± 0.67^∗∗^
Methanol extract	200	7.00 ± 0.35	11.34 ± 0.14^∗∗^

Results are stated as mean ± SEM (*n* = 5). ^*∗*^*P* < 0.05 and ^*∗∗*^*P* < 0.01, which are in contrast to the control group, are significant (one-way ANOVA followed by Dunnett's test).

**Table 4:** Sleeping time in mice was induced by the effect of leaves extract on thiopental sodium.

Group	Dose (mg/kg)	Latent period	Sleeping time	% effect
Control	10 mL/kg	11.0 ± 0.61	37.8 ± 2.35	0
Diazepam	0.5	2.8 ± 0.31	281.2 ± 14.22	743.92^∗∗^
Methanol extract	100	5.4 ± 0.71	148.6 ± 5.41	617.99^∗∗^
Methanol extract	200	4.2 ± 0.25	233.6 ± 6.55	680.45^∗∗^

Results are characterized as mean ± SEM (*n* = 5); ^*∗∗*^*P* < 0.01, that is in comparison with the control group significantly (one-way ANOVA followed by Dunnett's test).

**Table 5:** Potential neuropharmacological test of *L. coromandelica* leaves extract by the hole-cross test.

Group	Dose (mg/kg)	Quantity of moves (% of movements inhibition)				
		0 min	30 min	60 min	90 min	120 min
Control	10 mL/kg	6.0 ± 0.60	8.2 ± 0.77	5.8 ± 0.78	6.3 ± 0.70	9.6 ± 0.24
Standard	1	2.4 ± 0.82^∗∗^	4.0 ± 1.08	3.0 ± 1.00^*∗*^	2.9 ± 0.60^∗∗^	4.0 ± 0.63
Methanol extract	100	3.8 ± 1.15^∗∗^	3.2 ± 1.15^∗∗^	5.4 ± 0.67	5.4 ± 0.67	6.8 ± 0.58
Methanol extract	200	2.4 ± 0.70^∗∗^	3.1 ± 0.90^∗∗^	3.0 ± 0.48^∗∗^	3.6 ± 0.94^∗∗^	4.8 ± 0.81

Results are characterized as mean ± SEM (*n* = 5). ^*∗*^*P* < 0.05 and ^*∗∗*^*P* < 0.01, that is in comparison with the control group significantly (two-way ANOVA followed by Bonferroni's test).

**Table 6:** Neuropharmacological potential activity test using the hole-board test for leaves extracts.

Group	Dose (mg/kg)	Number of head dips	% inhibition
Control	10 mL/kg	22.4 ± 1.57	0
Standard	1	11.0 ± 0.70	50.89^∗∗∗^
Methanol extract	100	15.33 ± 0.83	31.56^∗∗^
Methanol extract	200	12.4 ± 0.71	44.64^∗∗∗^

Results are characterized as mean ± SEM (*n* = 5). ^*∗∗*^*P* < 0.01 and ^*∗∗∗*^*P* < 0.001, compared to the control group, are significant (one-way ANOVA followed by Bonferroni's test).

**Table 7:** Neuropharmacological potential activity test using the open-field test for the *L. coromandelica* leaves extract.

Group	Dose (mg/kg)	Number of movement (% of movements inhibition)				
		0 min	30 min	60 min	90 min	120 min
Control	10 mL/kg	26.8 ± 1.50	27.0 ± 2.38	28.6 ± 2.15	33.8 ± 2.03	34.6 ± 3.21
Standard	1	12.2 ± 3.23^*∗∗*^	15.8 ± 2.51^*∗∗*^	14.4 ± 3.36^*∗*^	14.0 ± 3.11^*∗∗*^	20.8 ± 3.48^*∗∗*^
Methanol extract	100	17.0 ± 1.52^*∗*^	19.0 ± 3.94^*∗*^	22.6 ± 2.11^*∗∗*^	27.0 ± 1.70^*∗*^	29.2 ± 2.98
Methanol extract	200	15.0 ± 2.02^*∗∗*^	17.0 ± 1.11^*∗∗*^	18.8.0 ± 1.42^*∗∗*^	21.0 ± 1.02^*∗*^	24.0 ± 1.09^*∗*^

Results are given as mean ± SEM (*n* = 5). ^*∗*^*P* < 0.05 and ^*∗∗*^*P* < 0.01, compared to the control group, are significant (two-way ANOVA followed by Bonferroni's test).

**Table 8:** Effects of *L. coromandelica* leaves extract on alloxan-induced diabetic mice's blood glucose levels.

Group	Dose (mg/kg)	Blood glucose level (mg/dl)				
		Day 0	Day 4	Day 7	Day 10	Day 15
Normal saline	0.3 ml	125.10 ± 2.50	108.10 ± 2.37	107.2 ± 0.48	104.30 ± 3.40	100.8 ± 2.24
Diabetic control	0.3 ml	420.40 ± 3.50	433.10 ± 2.37	439.25 ± 2.40	450.10 ± 2.58	466.20 ± 2.20
Glibenclamide	10	400.10 ± 3.37	377.20 ± 2.51^*∗*^	344.50 ± 2.19^*∗*^	301.70 ± 2.40^*∗∗*^	255.37 ± 3.10^*∗∗*^
Methanol extract	200	405.30 ± 3.51	396.15 ± 3.17	382.75 ± 2.40	371.10 ± 2.24	355.15 ± 3.30^*∗*^
Methanol extract	400	396.25 ± 3.57	378.55 ± 2.60	340.50 ± 2.44^*∗*^	318.10 ± 2.24^*∗∗*^	297.30 ± 3.32^*∗∗*^

Results are given as Mean ± SEM (*n* = 5). ^*∗*^*P* < 0.05 and ^*∗∗*^*P* < 0.01, compared to the control group, are significant (one-way ANOVA followed by Dunnett's test).

**Table 9:** Effects of *L. coromandelica* extracts on mice's body weight.

Group	Dose (mg/kg)	Body weight (g)				
		Day 0	Day 4	Day 7	Day 10	Day 15
Normal saline	0.3 ml	24.10 ± 0.37	26.10 ± 0.29	27.20 ± 0.48	28.50 ± 0.40	29.20 ± 0.24
Diabetic control	0.3 ml	28.25 ± 0.50	27.0 ± 0.07	26.25 ± 0.80	25.0 ± 0.48	24.20 ± 0.70
Glibenclamide	10	30.25 ± 0.33	31.75 ± 0.50	33.25 ± 0.45^*∗*^	34.25 ± 0.22^*∗∗*^	35.70 ± 0.30^*∗∗*^
Methanol extract	200	29.50 ± 0.65	29.95 ± 0.29	30.10 ± 0.15	31.85 ± 0.45	33.25 ± 0.50^*∗*^
Methanol extract	400	29.10 ± 0.25	30.25 ± 0.30	33.10 ± 0.44^*∗*^	33.75 ± 0.40^*∗*^	34.50 ± 0.25^*∗∗*^

Results are given as mean ± SEM (*n* = 5). ^*∗*^*P* < 0.05, compared to the control group, is significant (one-way ANOVA followed by Dunnett's test).

## Data Availability

The data used to support the findings of this study are included within the article.
